# An Overview of Alternating Electric Fields Therapy (NovoTTF Therapy) for the Treatment of Malignant Glioma

**DOI:** 10.1007/s11910-015-0606-5

**Published:** 2016-01-06

**Authors:** Kenneth D. Swanson, Edwin Lok, Eric T. Wong

**Affiliations:** Brain Tumor Center and Neuro-Oncology Unit, Beth Israel Deaconess Medical Center, Harvard Medical School, Boston, MA USA; Department of Physics, University of Massachusetts in Lowell, Lowell, MA USA

**Keywords:** Glioblastoma, Tumor treating fields, Malignant glioma, Septin, Tubulin, Computer modeling, Dexamethasone

## Abstract

As with many cancer treatments, tumor treating fields (TTFields) target rapidly dividing tumor cells. During mitosis, TTFields-exposed cells exhibit uncontrolled membrane blebbing at the onset of anaphase, resulting in aberrant mitotic exit. Based on these criteria, at least two protein complexes have been proposed as TTFields’ molecular targets, including α/β-tubulin and the septin 2, 6, 7 heterotrimer. After aberrant mitotic exit, cells exhibited abnormal nuclei and signs of cellular stress, including decreased cellular proliferation and p53 dependence, and exhibit the hallmarks of immunogenic cell death, suggesting that TTFields treatment may induce an antitumor immune response. Clinical trials lead to Food and Drug Administration approval for their treatment of recurrent glioblastoma. Detailed modeling of TTFields within the brain suggests that the location of the tumor may affect treatment efficacy. These observations have a profound impact on the use of TTFields in the clinic, including what co-therapies may be best applied to boost its efficacy.

## Introduction

Alternating electric fields therapy is a novel anticancer treatment that disrupts tumor cell mitosis. The first US Food and Drug Administration (FDA)-approved indication is for the treatment of recurrent glioblastoma. In this review, we will discuss the basic cell biology effects, physics properties, and clinical trial data of this emerging anticancer treatment.

## Cell Biology Effects of Tumor Treating Fields on Mitotic Tumor Cells

Electric fields have long been known to interact with biological material and this has been exploited for medical treatment by effecting the depolarization of electrically excitable nerves and muscles, or inducing the deep heating of tissues. Dr. Yorum Palti at the Rappaport Institute in Israel developed a technology to deliver electric fields that affect dividing cell viability. Analysis of this effect on cell viability revealed a tight peak of cytotoxic effect in all cell types tested between 150 and 200 kHz, which was not apparent at frequencies < 50 kHz and > 500 kHz. This cytotoxicity also increased with field intensity. Based on their ability to kill tumor cells in culture, these alternating electric fields have been referred to as tumor treating fields (TTFields) [1••, 2••]. During mitosis, exposure of cells to these fields results in violent membrane blebbing and cells exhibited disruption of microtubule spindle elements and chromosomal order post-mitosis [2••, 3••]. An enigmatic feature of the effect that TTFields have on cells is that the incident angle to the mitotic plate dictates the magnitude of cellular damage. When TTFields were perpendicular to the plane of division, cells were relatively unaffected but when the TTFields were parallel to the plane of division, cells exhibited a higher degree of mitotic failure [2••]. TTFields exposure perturbs both microtubule spindle elements and mitotic chromosomal order, demonstrating that cells are physically deranged post-mitosis. This suggests that TTFields interact with elements, likely a protein structure, within the cell that possesses a fixed structural relationship to the plane of division.

Detailed analysis has demonstrated that TTFields-induced mitotic disruption specifically occurs coincident with the exit from metaphase [3••]. Early reports showed that cells exposed to TTFields exhibited increase time in mitosis [2••]. Compared with paclitaxel treatment, which blocks metaphase exit, there was no gross perturbation in progression through mitosis [3••]. Microtubule structure during metaphase and both cyclin B and securin destruction was normal [4]. Time-lapse microscopy of mitotic cells stained with a vital DNA dye for staging of mitosis revealed that TTFields-induced membrane blebbing occurs at the expected time of metaphase exit [3••]. Since this is triggered by the capture of metaphase spindle microtubules by the kinetochores of chromosomes subsequent to metaphase plate formation (see below), this suggests that in the presence of TTFields metaphase spindle formation and function are normal. However, a measurable increase in the 4N DNA content of TTFields-treated cells combined with a persistence in phosphorylated histone H3 levels in synchronized cultures treated with TTFields indicated aberrant mitotic exit [3••, 4]. Together, these data demonstrate that TTFields-treated cells transit normally through metaphase but become deranged during anaphase due to the violent disruption of mitotic order secondary to membrane blebbing followed by aberrant mitotic exit.

TTFields have also been shown to cause tumor regression in animal models and human cancers. Treating mice with a number of injected tumor models, including CT26 colon adenocarcinoma, B16/F1 melanoma, Lewis lung carcinoma, and the highly invasive VX2 carcinoma in rabbits, all demonstrated TTFields-induced tumor regression [1••, 5, 6]. Interestingly, treatment of VX2 tumors implanted under the kidney capsule following tumor establishment led to a marked decrease in lung metastasis when analyzed 35 days after implantation. This was accompanied by a statistically significant increase in the number of immune infiltrates within successful lung metastases in treated animals. Together, these studies demonstrated that TTFields could penetrate the body and affect cellular physiology and lead to their testing against human gliomas and a successful phase III clinical trial (see below). Chen et al*.* [7] have also applied similar intermediate frequency alternating electric fields to B16/F10 melanoma cells, showing similar effects both *in vitro* and *ex vivo*. Interestingly, they also provide evidence that CD34-positive cell numbers were reduced, indicating an effect on the tumor microvasculature in the treated tumors [7]. Beyond being necessary for perfusion of oxygen and nutrients into the tumor bed, tumor endothelium has been implicated in supporting the intratumoral immune inhibitory environment [8, 9]. These data suggest that beyond their effects on the malignant cells, TTFields may contribute to tumor regression by both starving the tumor and reducing intratumoral immune privilege.

## Basis of Vulnerability to TTFields During Mitosis

The cell cycle is a regimented process that controls cellular growth and proliferation. Since TTFields affect cells during mitosis, this suggests a specific vulnerability within mitosis to TTFields-induced perturbation. Cellular growth and biomass accumulation occurs during interphase, which is subdivided into the G_1_, S, and G_2_ phases. Non-dividing and post-mitotic cells exist in a state referred to as G_0_. Cell division and daughter cell production occurs during mitosis, which is further subdivided into prometaphase, metaphase, anaphase, and telophase. In contrast to mitosis, the lengthy period of interphase is dominated by metabolic processes; by contrast, mitosis is characterized by processes that are dependent upon the rapid and precise assembling and functions of complex mitosis-specific structures. These structures are responsible for three independent, albeit coordinated, mechanical functions that are critical for proper cell division within a limited period of time, roughly 90 minutes in typical cultured cells. Therefore, while interphase is a highly anisotropic stage, the processes that mitosis depends on possess properties that make them inherently more susceptible to perturbation by electromotive forces introduced into the cell by TTFields (see below). In addition, as mitosis proceeds, both the level of order and the demand for spatial and temporal precision of execution by these structures crescendos from assembly of the mitotic spindles to the triggering of the significant biomechanical forces generated by the cytokinetic cleavage furrow (CCF) that is capable of dividing the parental cell into two daughters in only minutes. Therefore, as mitosis proceeds, cells are likely to be increasingly sensitive to the effects of alternating electric fields.

Sister chromatids that are condensed during prometaphase attach to the newly assembled metaphase spindles and migrate to the cell equator through the action of kinesin motor proteins to form the metaphase plate [10]. The kinetochores within the centromeric regions of each sister chromatid capture microtubule ends of the mitotic spindle. Since the kinetochores of each chromatid pair lays across the chromosomes from each other, microtubule capture insures that all chromatid pairs are aligned on the metaphase plate with their constituent kinetochores pairs aligned towards the respective pole of the each forming daughter cells. This also creates physical tension between the kinetochores, which acts to terminate a signal that prevents the activation of anaphase-promoting complex C (APC/C) and metaphase exit [11]. Final kinetochore capture triggers mitotic exit by permitting the rapid and irreversible activation of the APC/C ubiquitin ligase activity responsible for targeting specific proteins for destruction by the S26 proteosome. Destruction of APC/C ubiquinated substrates is necessary for metaphase exit, including G_2_ cyclin, cyclin B, and securin. Cyclin B is the allosteric activator of the cyclin-dependent kinase 1, whose activity both initiates mitosis and drives cells into metaphase while simultaneously inhibiting processes necessary for anaphase. Securin inhibits the protease separase that cleaves the cohesin protein complexes, which help to entangle sister chromatids prior to anaphase [12]. Since APC/C is only activated following the capture of the last kinetochore, the entry into anaphase therefore requires proper microtubule spindle formation and function [13].

**Fig. 1:**
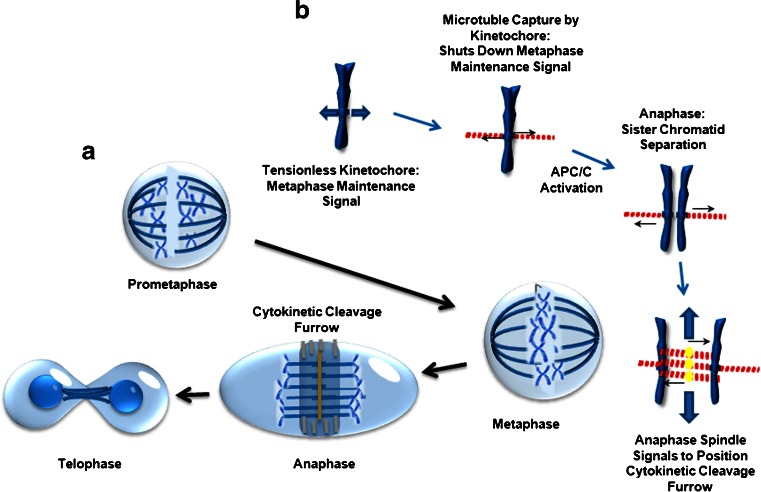
Tumor treating fields (TTFields) interact with cells during mitosis. The cell cycle can be separated into interphase and mitosis. Interphase is divided into G1, S, and G2, during which cells grow larger by accumulation of biomass through metabolic processes. Mitosis is divided into prometaphase, metaphase, anaphase, and telophase. These different phases are defined and dominated by biomechanical processes that have evolved to ensure the faithful inheritance of the parental genome in each newly formed daughter cell. TTFields affect cells within anaphase. APC/C = anaphase-promoting complex C

Within anaphase, two structures form, perform their function, and are then rapidly disassembled. The microtubular anaphase spindle performs two functions within minutes of anaphase entry. During this time, it is assembled between the separating daughter chromosomes, rapidly grow from the midline, and push them into the forming daughter cells. The midline of the anaphase spindle is essential for the organization and regulation of the CCF (Fig. 1). The combination of the precise coordination of events during anaphase and the short time frame in which they need to be executed likely makes them sensitive to the disruptive impacts of the TTFields. To accomplish this, the anaphase spindle midline contains a number of regulatory proteins, including the centralspindlin complex, RhoGEF, and ECT2, which recruits the adaptor protein anillin, which, in turn, binds to the septin 2, 6, 7 complex [14]. ECT2 is subsequently delivered from the midline to the CCF where it is instrumental in dictating its localization and regulating its contraction during cytokinesis [15]. Upon its recruitment to the CCF, the septin heterotrimers oligomerizes into a highly ordered cytoskeleton-like scaffold that functions to recruit and organize the actionmyosin contractile elements required for furrow ingression and the separation of the daughter cells [14, 16–20]. In addition to its function within the CCF, septins also cross-link F-actin bundles within the submembranous actin cytoskeleton [21, 22•, 23, 24]. This structure must possess adequate rigidity to withstand the hydrostatic pressures within the cytoplasm generated by ingression of the cytokinetic furrow. Failure to restrain these forces leads to rupture of the connection between this structure and the overlying plasma membrane, leading to membrane blebbing [21].

## Molecular Targets of TTFields

The disruption of cells by TTFields during mitosis suggests that they exert forces or movement on definable molecular targets, the functions of which are critical to a mitotic process or processes. Proteins possess complex charge structures on their surfaces that are dependent on the charges of surface amino acid side chains. The arrangement of acidic and basic side chains can result in regional separations of surface charge imparting a dipole moment onto the protein, similar to that observed in bar magnets. The dipole moments of such proteins will align within an electric field to orient towards the oppositely charged pole of the fields. Therefore, the re-polarization of the alternating field will induce a re-alignment of the protein dipoles within the field. Thus, such proteins would be expected to experience rotational forces within TTFields [2••].

Proteins that are important to mitotic progression that have high dipole moments have been suggested to be targets of TTFields perturbation, include the α/β-tubulin monomeric subunit of microtubule and the mitotic septin complex. α/β-Tubulin form the building blocks for microtubules. Taxanes are commonly used chemotherapeutic agents that bind and stabilize microtubules and can cause mitotic catastrophe by trapping cells within metaphase [25]. The functional subunit of microtubules is a heterodimer consisting of α- and β-tubulin, which possesses a high predicted dipole moment of 1660 Debyes (D) (PDB 1JFF) [26, 27]. Therefore, it is possible that TTFields interfere with a critical mitotic function performed by microtubules [1••, 2••], including the formation of the metaphase and anaphase spindles and their respective mechanical functions [28, 29], or the astral microtubules that help regulate the CCF [30].

The septin 2, 6, 7 heterotrimer also possesses a high predicted dipole moment of 2711 D (PDB 2QAG), [27, 31]. As described above, the septin 2, 6, 7 heterotrimeric complex is required for functions that are necessary for the later stages of cell division. The complex rapidly polymerizes and structurally helps to organize the CCF during anaphase. Once recruited, it then oligomerizes and organizes the CCF above the equatorial cleavage plane by binding to F-actin filaments and spatially regulates myosin activation. The perturbation of the septin complex is particularly enticing because of its known roles in the regulation of CCF function and actin bundle cross-linking and organization of structures such as the cellular submembranous actin cytoskeleton that is required for its rigidity [21, 22•]. Short hairpin RNA-driven depletion of septin 7 resulted in mitotic blebbing with similarities to that seen with TTFields treatment [2••, 3••, 22•], and an increase in cell size [22•, 32]. Septins also interact with microtubules and several microtubule interacting proteins that influence their position and stability during both interphase and mitotsis [33]. Therefore, perturbation of either α/β-tubulin or septins may perturb microtubule function. Unlike errors or damage that initiate the G_1_/S, G_2_/M or spindle assembly check point (SAC), catastrophic errors occurring after the cell anaphase commitment are unlikely to be corrected [34]. These observations strongly suggests a mechanism of action where TTFields perturb mitosis by interfering with normal septin localization and function during mitosis, leading membrane blebbing and aberrant mitotic exit.

## Mitotic Effects of TTFields Result in Postmitotic Stress

This aberrant mitotic exit results in a high degree of cellular stress, as indicated by increased cytoplasmic vacuoles, as well as decrease in proliferation and apoptosis [3••]. Cells experiencing mitotic exit in the absence of division have been shown to experience p53-dependent G_0/1_ cell cycle arrest and apoptosis [35, 36]. Likewise, cells exposed to TTFields subsequently exhibit decreased proliferation with a failure to enter S phase and increased levels of apoptosis beginning > 24 hours after TTFields exposure in a p53-dependent manner. Apoptosis occurs after 24 hours of TTFields exposure during mitosis in a p53-dependent manner [3••]. These data strongly suggest that the efficacy of treatment may be influenced by tumor genetics.

There are different ways in which TTFields may affect patient outcomes. TTFields are able to disrupt cells during mitosis and this leads to aberrant mitotic exit and cell death. As in the case of spindle poisons, which trigger the SAC, cells affected by TTFields exhibit different fates, including death in anaphase or aberrant exit from mitosis similar to mitotic slippage. In this way, the mechanism of action may be similar to that proposed for other cancer therapies that destroy tumor cells based on their increased proliferation rate making them more susceptible to agents that target dividing cells, such as spindle poisons. Alternatively, there are multiple lines of evidence suggesting that TTFields induce an immunological response against tumors.

Several lines of evidence support a possible immune dependency for TTFields efficacy. Senovilla *et al.* showed that tetraploid cells that are produced under experimental conditions that perturb mitotic exit exhibit the hallmarks of immunogenic cell death (ICD) [37]. This programmed form of cell death evokes an immune response against the dying cells through cell surface expression of the endoplasmic reticulum chaperone protein, calreticulin, and the secretion of the cytokine/alarmin, high mobility group box 1 protein (HMGB1), and adenosine triphosphate [38••, 39]. When injected into mice, these dying cells produced a protective immunization against subsequent challenge with the same tumor cells [37]. Additionally, it has been demonstrated that cells made tetraploid by pharmacologic manipulation also express natural killer group 2, member D (NKG2D) and DNAX accessory molecule 1 (DNAM) ligands on their surfaces, which provoke natural killer cell clearance of the expressing cells [40]. Cells that are exposed to TTFields exhibit cellular responses that are consistent with ICD, including the cell surface expression of calreticulin and depletion of HMGB1. Kirson et al. [5] showed that a brief TTFields treatment of subrenal capsule-injected VX2 tumor in rabbits markedly reduced subsequent metastatic spread to the lungs. Examination of metastatic tumors in the lungs of these TTFields-treated rabbits showed a significant increase in immune infiltrates, likely indicating a requirement for increased immune protective stroma for tumors capable of developing in these animals [5]. In the pivotal EF-11 trial that lead to FDA approval for the treatment of recurrent glioblastoma, response typically occurred 6.6–9.9 months following the onset of treatment, at which point responders exhibited rapid tumor regression [41•]. This pattern of delayed response is also consistent with an immune mechanism of tumor rejection. Finally, clinical data strongly suggest that concurrent use of dexamethasone, a potent immunosuppressive agent, is correlated with poor outcome (see below) [41•, 42••].

## TTFields Therapy for Recurrent Glioblastoma

The current FDA-approved indication for the TTFields therapy device is treatment of recurrent glioblastoma. The first-in-human pilot trial for the safety and efficacy of TTFields therapy was conducted in 2004 to 2007 and enrolled 10 patients with recurrent glioblastoma [1••]. The most common adverse event was contact dermatitis, which occurred in nine patients as a result of hydrogel-induced irritation of the scalp. Two patients experienced partial seizures that were related to their tumors. No toxicity on blood count or chemistry was seen, except for elevated liver enzymes in those taking anticonvulsants. The median overall survival (mOS) of the 10 patients was 14.4 months. The time to tumor progression was 6.0 months and the 1-year survival rate was 67.5 % [1••]. There was one complete and one partial responder who were alive at 84 and 87 months, respectively, from treatment initiation [43]. Furthermore, the intensity of electric fields as directly measured in one patient was validated to be within 10 % of the values estimated by computer modeling [1••].

The phase III registration trial was conducted in 2006 to 2009 and the primary end point was overall survival [44••]. In the intent-to-treat population, the mOS was 6.6 months for TTFields versus 6.0 months for best physician’s choice (BPC) chemotherapy, with a hazard ratio (HR) of 0.86 (*p* = 0.27). About 31 % of the BPC cohort received bevacizumab alone or in combination with chemotherapy. The median progression-free survival (PFS) of TTFields and BPC chemotherapy was 2.2 and 2.1 months, respectively (HR 0.81; *p* = 0.16), and the PFS at 6 months was 21.4 % and 15.1 %, respectively (*p* = 0.13). One-year survival rate was 20 % in both cohorts. The outcome of the trial indicates that TTFields probably has equivalent efficacy when compared with chemotherapy and bevacizumab.

Grade 1 or 2 scalp irritation were the most common adverse events associated with the device. Shifting of the arrays slightly during array exchange and by applying topical corticosteroid can minimize this irritation [45]. There was far less hematological toxicity, appetite loss, constipation, diarrhea, fatigue, nausea, vomiting, and pain associated with the device when compared to BPC chemotherapy. Furthermore, analysis showed that device-treated patients had better cognitive and emotional functions. Based on the equivalent efficacy results and absence of serious associated toxicities, the FDA approved on 8 April 2011 the TTFields therapy for the treatment of recurrent glioblastoma.

The apparent discrepancy in the overall survival rates between the pilot study and the registration trial prompted a series of post hoc analyses of the trial data. First, one of the analyses centered on responders and it showed that five of 14 responders treated with TTFields monotherapy had prior low-grade histology, while none of the seven responders treated with BPC chemotherapy did [41•]. Second, the analysis revealed significantly less dexamethasone use in responders versus nonresponders [41•]. Responders in the TTFields monotherapy group received a median dexamethasone dose of 1.0 mg/day while nonresponders received 5.2 mg/day. A similar difference was also noted in the median cumulative dexamethasone dose of 7.1 mg for responders versus 261.7 mg for nonresponders. In the chemotherapy cohort, the median dexamethasone dose was 1.2 mg/day for responders versus 6.0 mg/day for nonresponders. However, the median cumulative dexamethasone dose was not significantly different (348.5 mg for responders vs 242.3 mg for nonresponder). These data suggest that TTFields efficacy may be influenced by concurrent dexamethasone use, which is a clinically modifiable factor. This finding prompted an in-depth analysis of the dexamethasone effect in the entire trial population.

Applying an unsupervised modified binary search algorithm that stratified the TTFields monotherapy arm of the phase III trial based on the dexamethasone dosage that provided the greatest statistical difference in survival revealed that subjects who used > 4.1 mg/day dexamethasone had a markedly shortened mOS of 4.8 months compared with those who received ≤ 4.1 mg/day (mOS of 11.0 months) [42••]. Patients in the chemotherapy arm were observed to have a similar but less robust dichotomization; those who used > 4.1 and ≤ 4.1 mg/day dexamethasone had a mOS of 6.0 and 8.9 months, respectively. This difference in overall survival based on dexamethasone dose was unrelated to tumor size but most likely from interference with patient immune effector function. A single institution validation cohort of patients treated with TTFields therapy, using their CD3^+^, CD4^+^, and CD8^+^ T lymphocytes as a marker of immune competency, suggested the importance of immune competence to TTFields therapy. Importantly, a dexamethasone dosage of > 4.0 mg/day was also found to be a poor prognostic factor in newly diagnosed patients who completed radiotherapy [46], supporting the conclusion that dexamethasone can interfere with treatment. With successive increases in dexamethasone dosage, both cohorts reached an inflection point near 8.0 mg/day, after which the rate of survival decreased slowly thereafter. Taken together, dexamethasone exerts a generalized and profound interference on the efficacy of both TTFields and chemotherapeutic treatment against glioblastoma. Therefore, dexamethasone use should be minimized [47].

## Transcanial Distribution of Electric Fields from Transducer Arrays

**Fig. 2:**
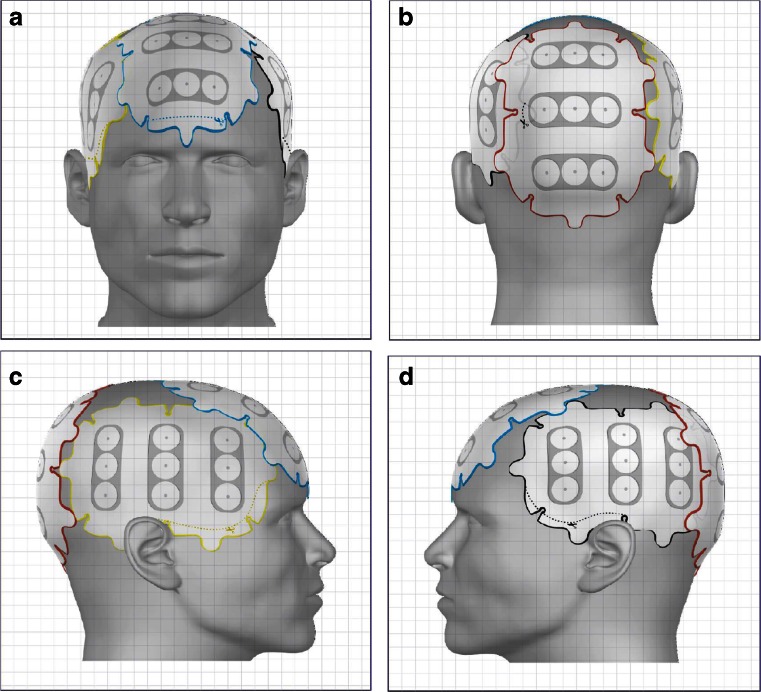
Transducer array placement on the scalp. As generated by the mapping software NovoTAL, two pairs of arrays are positioned orthogonally in an anterior–posterior (A, B), as well as right–left (C, D), arrangement. These two pairs of arrays are connected to an electric field generator, which is, in turn, either connected to a portable battery pack or a power cord that can be inserted directly into an electric outlet (not shown)

**Fig. 3:**
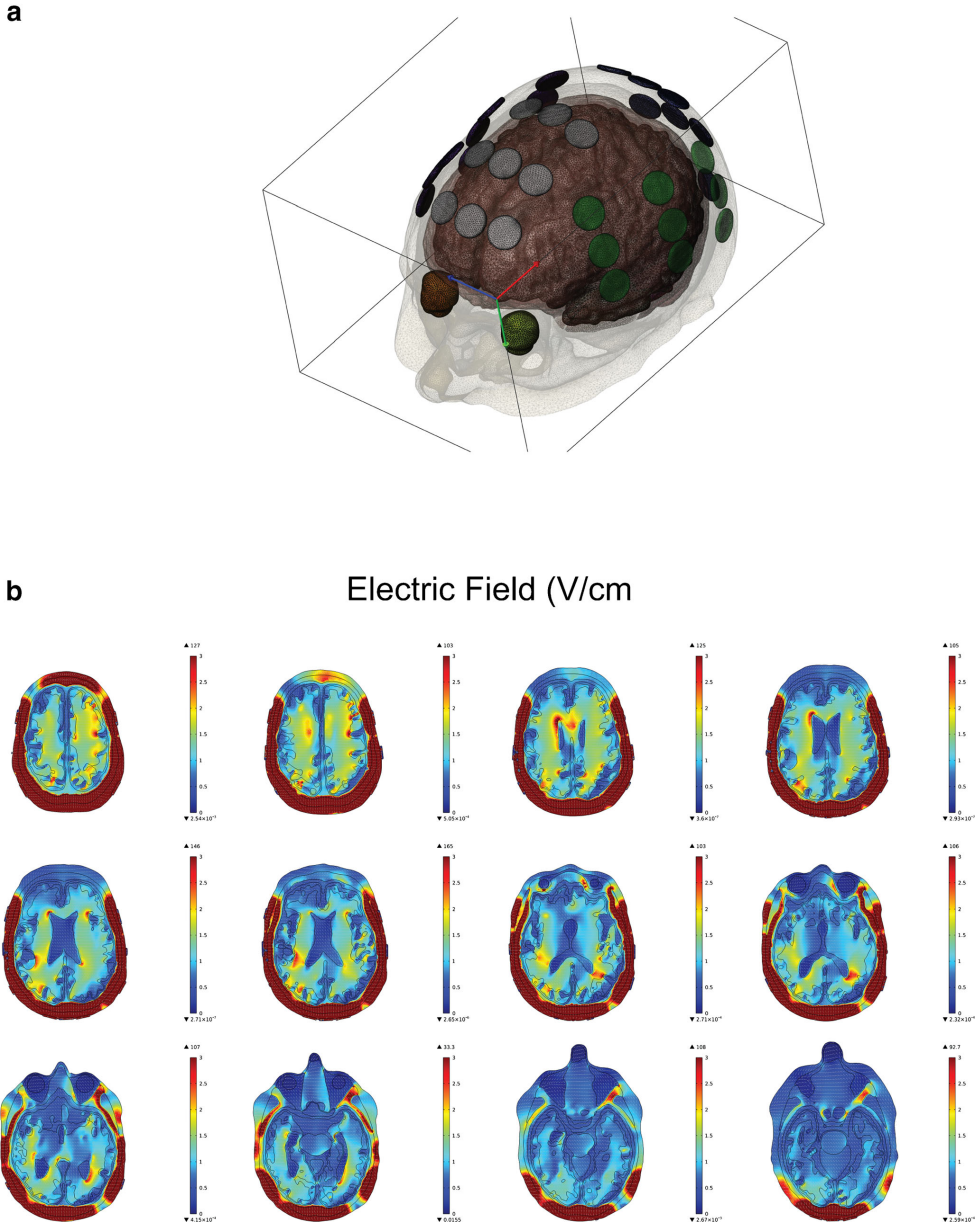
Computer modeling of electric field distribution within the brain. T1-weighed postgadolinium, T2, and magnetization-prepared rapid gradient-echo magnetic resonance images are imported into Simpleware’s ScanIP 7.0 Suite to perform segmentation of various brain structures, including the scalp, skull, dura, cerebrospinal fluid, gray matter, white matter, brainstem, cerebellum, bilateral ventricles, gross tumor volume, and tumor necrotic core. (A) An air-tight volumetric mesh is then generated for finite element analysis using COMSOL Multiphysics. (B) The distribution of electric fields within the brain is inhomogeneous, with the highest fields at the frontal and occipital horns of the lateral ventricles, as well as the medial surface of the glioblastoma

A number of factors, including a medium’s electric conductivity and relative permittivity, can affect electric field distrubution. Since each tissue composition is unique, the intracranial structures must therefore be characterized based on their conductivity and permittivity values. The highly heterogeneous architecture of the brain therefore distort electric fields induced by an external source. Electric fields are generally defined as instantaneous changes in electric potential. This change in electric potential results in electromotive disruption of mitotic structures and is therefore the basis for the therapeutic benefit of TTFields [3••]. TTFields therapy for glioblastoma is delivered by two pairs of transducer arrays positioned orthogonally on the shaved scalp, adhered by a thin layer of conductive gel that provides good conductivity (Fig. 2) [48] . TTFields are generated by a battery-powered alternating current generator, operating at 200 kHz, with maximum voltage alternating from +50 to –50 V. To obtain a comprehensive model of the electric fields distribution in the brain, computer modeling can be performed using co-registered patient Digital Imaging and Communications in Medicine (DICOM) datasets from T1-weighed postgadolinium, T2, and magnetization-prepared rapid gradient-echo magnetic resonance images. Previously, Lok et al. [49•] have shown a heterogeneous distribution of electric fields in the brain, and the regions adjacent to the ventricular horns had a particularly high electric field intensity (Fig. 3). This is likely due to the higher electric conductivity of cerebrospinal fluid (CSF) than the surrounding tissues, which behaves like the terminal of a capacitor, with the surrounding tissues functioning much like a dielectric between conductive terminals. Since a dielectric medium generally retains charge, the rate at which the medium is able to collect and retain the charge is defined by its conductivity and relative permittivity. At 200 kHz, the effect of permittivity is overwhelmed by the conductivity of the medium [50]. Furthermore, the rate at which the medium is able to collect and retain charges is frequency dependent. At high frequencies, each medium has a unique capacitive reactance characteristic of the medium’s conductivity, and thus the medium only has limited time to collect a finite amount of charge and retain it before the field collapses as the polarity changes direction, thereby discharging the initially retained charge before repeating the process. Since CSF has a low permittivity value compared with its surrounding tissues, it is a poor dielectric medium and thus charges will migrate through the fluid layer at a much faster rate with minimal charge retention. This explains why most of the CSF exhibits very low electric field intensity. However, this is not true at the interface between CSF and its adjacent brain tissue. The computed electric field distribution revealed that the ventricular horns exhibit a higher electric field intensity than the rest of the CSF space. This is likely due to the geometry of the region coupled with increased electric potential and reactance causing large field changes.

The electric properties of gliomas are likely to vary among patients, depending on their tumor composition. Tumors with larger necrotic cores are likely to exhibit higher field intensities in the gross tumor volume owing to the capacitive reactance as explained above. In contrast, tumors with smaller or no necrotic core will likely exhibit lower field intensities at the center of the volume due to absence of a conductive medium to act as an electric current source. This may become clinically relevant owing to the increased requirement for time of exposure to TTFields as the outer layers of the gross tumor volume is treated slowly because of lower field intensities.

## The Use of TTFields Therapy in Clinical Practice

The post-FDA-approved use of TTFields therapy in routine clinical practice may differ from that in the registration trial because of the stringent entry criteria built into the trial. Therefore, a Patient Registry Dataset (PRiDe) was developed in an effort to capture pertinent clinical practice data. This dataset consisted of 457 patients from 91 treatment centers in the US. Patients treated and captured in PRiDe had a mOS of 9.6 months compared with the 6.6 months in the TTFields monotherapy arm in the registration trial [44••, 51]. The 1-year OS rate was also longer at 44 % compared with 20 %, respectively [44••, 51]. The difference in survival characteristics is most likely due to the higher proportion of patients treated with TTFields at first recurrence in PRiDe (33 %) than that in the registration trial (9 %). Treatment at an earlier time point in the process of disease progression may provide a higher efficacy than treatment at a later time point. Absence of prior bevacizumab usage was also favorable [51]. However, the heterogeneity in the adjunctive treatments used in conjunction with TTFields therapy in the PRiDe dataset, which included cytotoxic chemotherapy, bevacizumab, or even alternative medicine that were not adequately captured, is an important caveat that makes it statistically noncomparative with the TTFields monotherapy arm in the registration trial.

## Efficacy of TTFields Therapy for Newly Diagnosed Glioblastoma

TTFields therapy is currently being tested in glioblastoma patients after their initial radiotherapy and concomitant daily temozolomide. In this phase III trial, 700 patients were randomized 2:1 to received either TTFields plus adjuvant temozolomide or temozolomide alone, respectively [52, 53]. The primary end point was PFS. In a prespecified interim analysis of the first 315 patients after a minimum follow-up of 18 months, the intent-to-treat cohort that received TTFields plus temozolomide had a longer PFS than the cohort treated with temozolomide alone (median 7.1 vs 4.0 months, HR 0.6; log-rank *p* = 0.0014). The mOS also favors the TTFields plus temozolomide group (19.6 vs 16.6 months, HR 0.75; log-rank *p* = 0.034), as well as the per-protocol population that started the second cycle of treatment (20.5 vs 15.5 months, HR 0.67; log-rank *p* = 0.0072).

The trial population had no unexpected adverse events. Grade 3 and 4 hematological toxicities between the TTFields plus temozolomide and temozolomide alone cohorts (12 % vs 9 %), gastrointestinal disorders (5 % vs 2 %), and convulsions (7 % vs 7 %) were not significantly different. Only scalp reaction was more common than those that had temozolomide only.

## Conclusion and Future Directions

TTFields interferes with α-/β-tubulin and septin 2, 6, 7 heterotrimer function in tumor cells during mitosis. A phase III clinical trial has shown a favorable toxicity profile in recurrent glioblastoma and promising efficacy data in newly diagnosed glioblastoma. Computer modeling showed inhomogeneous distribution of electric fields within the brain. Future investigations will likely include combination treatments, including immune therapies, that can potentially boost the existing efficacy of TTFields monotherapy.

## References

[1.••] Kirson ED, Dbaly V, Tovarys F, Vymazal J, Soustiel JF, Itzhaki A (2007). Alternating electric fields arrest cell proliferation in animal tumor models and human brain tumors. Proc Natl Acad Sci U S A.

[2.••] Kirson ED, Gurvich Z, Schneiderman R, Dekel E, Itzhaki A, Wasserman Y (2004). Disruption of cancer cell replication by alternating electric fields. Cancer Res.

[3.••] Gera N, Yang A, Holtzman TS, Lee SX, Wong ET, Swanson KD (2015). Tumor treating fields perturb the localization of septins and cause aberrant mitotic exit. PLoS ONE.

[4] Lee SX, Wong ET, Swanson KD (2013). Disruption of cell division within anaphase by tumor treating electric fields (TTFields) leads to immunogenic cell death. Neurooncology.

[5] Kirson ED, Giladi M, Gurvich Z, Itzhaki A, Mordechovich D, Schneiderman RS (2009). Alternating electric fields (TTFields) inhibit metastatic spread of solid tumors to the lungs. Clin Exp Metastasis.

[6] Kirson ED, Schneiderman RS, Dbaly V, Tovarys F, Vymazal J, Itzhaki A (2009). Chemotherapeutic treatment efficacy and sensitivity are increased by adjuvant alternating electric fields (TTFields). BMC Med Phys.

[7] Chen H, Liu R, Liu J, Tang J (2012). Growth inhibition of malignant melanoma by intermediate frequency alternating electric fields, and the underlying mechanisms. J Int Med Res.

[8] Lu J, Ye X, Fan F, Xia L, Bhattacharya R, Bellister S (2013). Endothelial cells promote the colorectal cancer stem cell phenotype through a soluble form of Jagged-1. Cancer Cell.

[9] Feng L, Sun X, Csizmadia E, Han L, Bian S, Murakami T (2011). Vascular CD39/ENTPD1 directly promotes tumor cell growth by scavenging extracellular adenosine triphosphate. Neoplasia.

[10] Zhu C, Zhao J, Bibikova M, Leverson JD, Bossy-Wetzel E, Fan JB (2005). Functional analysis of human microtubule-based motor proteins, the kinesins and dyneins, in mitosis/cytokinesis using RNA interference. Mol Biol Cell.

[11] Nezi L, Musacchio A (2009). Sister chromatid tension and the spindle assembly checkpoint. Curr Opin Cell Biol.

[12] Oliveira RA, Hamilton RS, Pauli A, Davis I, Nasmyth K (2010). Cohesin cleavage and Cdk inhibition trigger formation of daughter nuclei. Nat Cell Biol.

[13] Ge S, Skaar JR, Pagano M (2009). APC/C- and Mad2-mediated degradation of Cdc20 during spindle checkpoint activation. Cell Cycle.

[14] Field CM, Coughlin M, Doberstein S, Marty T, Sullivan W (2005). Characterization of anillin mutants reveals essential roles in septin localization and plasma membrane integrity. Development.

[15] Gregory SL, Ebrahimi S, Milverton J, Jones WM, Bejsovec A, Saint R (2008). Cell division requires a direct link between microtubule-bound RacGAP and Anillin in the contractile ring. Curr Biol.

[16] Straight AF, Field CM, Mitchison TJ (2005). Anillin binds nonmuscle myosin II and regulates the contractile ring. Mol Biol Cell.

[17] Piekny AJ, Glotzer M (2008). Anillin is a scaffold protein that links RhoA, actin, and myosin during cytokinesis. Curr Biol.

[18] Frenette P, Haines E, Loloyan M, Kinal M, Pakarian P, Piekny A (2012). An anillin-Ect2 complex stabilizes central spindle microtubules at the cortex during cytokinesis. PLoS ONE.

[19] Giansanti MG, Bonaccorsi S, Gatti M (1999). The role of anillin in meiotic cytokinesis of *Drosophila* males. J Cell Sci.

[20] Goldbach P, Wong R, Beise N, Sarpal R, Trimble WS, Brill JA (2010). Stabilization of the actomyosin ring enables spermatocyte cytokinesis in *Drosophila*. Mol Biol Cell.

[21] Gilden J, Krummel MF (2010). Control of cortical rigidity by the cytoskeleton: emerging roles for septins. Cytoskeleton (Hoboken).

[22.•] Gilden JK, Peck S, Chen YC, Krummel MF (2012). The septin cytoskeleton facilitates membrane retraction during motility and blebbing. J Cell Biol.

[23] Tooley AJ, Gilden J, Jacobelli J, Beemiller P, Trimble WS, Kinoshita M (2009). Amoeboid T lymphocytes require the septin cytoskeleton for cortical integrity and persistent motility. Nat Cell Biol.

[24] Hagiwara A, Tanaka Y, Hikawa R, Morone N, Kusumi A, Kimura H (2011). Submembranous septins as relatively stable components of actin-based membrane skeleton. Cytoskeleton (Hoboken).

[25] Yvon AM, Wadsworth P, Jordan MA (1999). Taxol suppresses dynamics of individual microtubules in living human tumor cells. Mol Biol Cell.

[26] Lowe J, Li H, Downing KH, Nogales E (2001). Refined structure of alpha beta-tubulin at 3.5 A resolution. J Mol Biol.

[27] Felder CE, Prilusky J, Silman I, Sussman JL (2007). A server and database for dipole moments of proteins. Nucleic Acids Res.

[28] Albertson R, Cao J, Hsieh TS, Sullivan W (2008). Vesicles and actin are targeted to the cleavage furrow via furrow microtubules and the central spindle. J Cell Biol.

[29] D'Avino PP, Savoian MS, Glover DM (2005). Cleavage furrow formation and ingression during animal cytokinesis: a microtubule legacy. J Cell Sci.

[30] Rankin KE, Wordeman L (2010). Long astral microtubules uncouple mitotic spindles from the cytokinetic furrow. J Cell Biol.

[31] Sirajuddin M, Farkasovsky M, Hauer F, Kuhlmann D, Macara IG, Weyand M (2007). Structural insight into filament formation by mammalian septins. Nature.

[32] Giladi M, Schneiderman RS, Porat Y, Munster M, Itzhaki A, Mordechovich D (2014). Mitotic disruption and reduced clonogenicity of pancreatic cancer cells in vitro and in vivo by tumor treating fields. Pancreatology.

[33] Bowen JR, Hwang D, Bai X, Roy D, Spiliotis ET (2011). Septin GTPases spatially guide microtubule organization and plus end dynamics in polarizing epithelia. J Cell Biol.

[34] Huang HC, Shi J, Orth JD, Mitchison TJ (2009). Evidence that mitotic exit is a better cancer therapeutic target than spindle assembly. Cancer Cell.

[35] Margolis RL, Lohez OD, Andreassen PR (2003). G1 tetraploidy checkpoint and the suppression of tumorigenesis. J Cell Biochem.

[36] Ganem NJ, Pellman D (2007). Limiting the proliferation of polyploid cells. Cell.

[37] Senovilla L, Vitale I, Martins I, Tailler M, Pailleret C, Michaud M (2012). An immunosurveillance mechanism controls cancer cell ploidy. Science.

[38.••] Kepp O, Senovilla L, Vitale I, Vacchelli E, Adjemian S, Agostinis P (2014). Consensus guidelines for the detection of immunogenic cell death. Oncoimmunology.

[39] Kepp O, Tesniere A, Schlemmer F, Michaud M, Senovilla L, Zitvogel L (2009). Immunogenic cell death modalities and their impact on cancer treatment. Apoptosis.

[40] Acebes-Huerta A, Lorenzo-Herrero S, Folgueras AR, Huergo-Zapico L, Lopez-Larrea C, Lopez-Soto A, Gonzalez S. Drug-induced hyperploidy stimulates an anti-tumor NK cell response mediated by NKG2D and DNAM-1 receptors. Oncoimmunology 2015. doi:10.1080/2162402X/2015.1074378. 10.1080/2162402X.2015.1074378 PMC4801427 27057443

[41.•] Wong ET, Lok E, Swanson KD, Gautam S, Engelhard HH, Lieberman F (2014). Response assessment of NovoTTF-100A versus best physician's choice chemotherapy in recurrent glioblastoma. Cancer Med.

[42.••] Wong ET, Lok E, Gautam S, Swanson KD (2015). Dexamethasone exerts profound immunologic interference on treatment efficacy for recurrent glioblastoma. Br J Cancer.

[43] Rulseh AM, Keller J, Klener J, Sroubek J, Dbaly V, Syrucek M (2012). Long-term survival of patients suffering from glioblastoma multiforme treated with tumor-treating fields. World J Surg Oncol.

[44.••] Stupp R, Wong ET, Kanner AA, Steinberg D, Engelhard H, Heidecke V (2012). NovoTTF-100A versus physician's choice chemotherapy in recurrent glioblastoma: a randomised phase III trial of a novel treatment modality. Eur J Cancer.

[45] Lacouture ME, Davis ME, Elzinga G, Butowski N, Tran D, Villano JL (2014). Characterization and management of dermatologic adverse events with the NovoTTF-100A System, a novel anti-mitotic electric field device for the treatment of recurrent glioblastoma. Semin Oncol.

[46] Back MF, Ang EL, Ng WH, See SJ, Lim CC, Chan SP (2007). Improved median survival for glioblastoma multiforme following introduction of adjuvant temozolomide chemotherapy. Ann Acad Med Singap.

[47] Rutz HP, Hofer S, Peghini PE, Gutteck-Amsler U, Rentsch K, Meier-Abt PJ (2005). Avoiding glucocorticoid administration in a neurooncological case. Cancer Biol Ther.

[48] McAdams ET, Jossinet J, Lackermeier A, Risacher F (1996). Factors affecting electrode-gel-skin interface impedance in electrical impedance tomography. Med Biol Eng Comput.

[49.•] Lok E, Hua V, Wong ET (2015). Computed modeling of alternating electric fields therapy for recurrent glioblastoma. Can Med.

[50] Ramos A, Morgan H, Green NG, Castellanos A (1998). Ac electrokinetics: a review of forces in microelectrode structures. J Phys D Appl Phys.

[51] Mrugala MM, Engelhard HH, Dinh Tran D, Kew Y, Cavaliere R, Villano JL (2014). Clinical practice experience with NovoTTF-100A system for glioblastoma: The Patient Registry Dataset (PRiDe). Semin Oncol.

[52] Stupp R, Wong E, Scott C, Taillibert S, Kanner A, Kesari S (2014). NT-40 interim analysis of the EF-14 trial: a prospective, multi-center trial of NovoTTF-100A together with temozolomide compared to temozolomide alone in patients with newly diagnosed GBM. Neurooncology.

[53] Stupp R, Taillibert S, Kanner AA, Kesari S, Steinberg DM, Toms SA, et al. Maintenance therapy with tumor-treating fields plus temozolomide vs temozolomide alone for glioblastoma: a randomized clinical trial. JAMA 2015;314:2535–2543. 10.1001/jama.2015.16669 26670971

